# Predicting how US public opinion on moral issues will change from 2018 to 2020 and beyond

**DOI:** 10.1098/rsos.211068

**Published:** 2022-04-13

**Authors:** Pontus Strimling, Irina Vartanova, Kimmo Eriksson

**Affiliations:** ^1^ Institute for Futures Studies, Box 591, 101 31 Stockholm, Sweden; ^2^ School of Education, Culture and Communication, Mälardalen University, Box 883, 721 23 Västerås, Sweden

**Keywords:** public opinion change, moral foundations, predictions, general social survey, Moral argument theory

## Abstract

The General Social Survey, conducted every 2 years, measures public opinion on a wide range of moral issues. The data from the 2020 survey are expected to be released in mid-October 2021. In advance of this data release, we make predictions for how public opinion will have shifted since 2018. We also predict further public opinion shifts for the coming decade up until the year 2030. These predictions are based on the theory that an opinion will become more popular over time if it holds an argument advantage, that is, if it is better justified by generally accepted kinds of arguments than the opposite opinion is. While this theory has successfully accounted for historical opinion trends, this is the first time it is used to predict future shifts. To assess the accuracy of our forecast we will compare it with the benchmark forecast that predicts the same public opinion in 2020 as in 2018.

## 1. Introduction

The ‘culture war’ is an expression from a book by James David Hunter [1]. It refers to a conflict between the moral values of conservatives and liberals in the United States, fought over non-economic policy issues such as women's rights, gay rights, civil rights, and gun rights. Opinions on such moral issues are not set in stone, however. Gay rights is a famous example of a moral issue where public opinion has shifted dramatically [2], but less dramatic opinion trends in the liberal direction over the last 40–50 years have been observed for a wide range of moral issues [3]. A theory that attempts to account for these opinion trends is the moral argument theory of Eriksson & Strimling [4]. This theory is based on four assumptions:

(1) In their daily life, people are exposed to arguments for different opinions, either from other people or from the media.
(2) Some kinds of moral arguments are generally acceptable, that is, relevant to the moral judgements of both liberals and conservatives. Other kinds of moral arguments are only relevant to conservatives and, therefore, not generally acceptable.
(3) For any specific moral opinion, there is general agreement that only certain kinds of moral arguments can be used to justify it. On any given issue, one opinion will, therefore, have an ‘argument advantage’ in the sense that this opinion is easier to justify by generally acceptable kinds of arguments than the opposite opinion is.
(4) People are more likely to change opinion when confronted with an argument of a kind that is relevant to their moral judgements.

Under these assumptions, the theory concludes that by measuring which opinion on an issue has the argument advantage, and how large the advantage is, we should be able to predict in which direction, and how fast, public opinion will change. Specifically, the higher the argument advantage of an opinion is, the faster it should gain in popularity. This verbal argument is also supported by computational models [4,5].

There is empirical evidence for each of the four assumptions behind the theory. The first assumption, that people are exposed to arguments on moral issues, is supported by media content analyses [6,7]. The second assumption, about the acceptability of different kinds of arguments, is supported by moral foundations theory. Moral foundations theory distils moral arguments into just a few kinds [8]. Extensive studies have asked people how relevant these kinds of arguments are for their moral judgements [9,10]. According to these studies, conservatives find all kinds of arguments relevant while liberals only find those kinds of arguments relevant that are generally acceptable (harm, violence, fairness and liberty). The third assumption, that there is general agreement about which kinds of arguments can be used to justify which opinion on an issue, is supported by multiple studies conducted in the United States, the United Kingdom, Brazil and Israel [5,11]. The fourth assumption, that opinion change from argument exposure is linked to how acceptable that kind of argument is to a person, is supported by experimental studies [12].

The validity of the theory is supported by recent studies in the United States and the United Kingdom, in which recent historical opinion trends for a large number of specific moral issues have been predicted by measures of which opinion on each issue has the argument advantage [5,10]. The goal of this project is to test if the moral argument theory is also capable of predicting *future* opinions. Specifically, we will use measurements of argument advantage to predict how public opinion in the United States will move on a wide range of moral issues.

Note that such predictions can never be perfectly accurate. One reason is that opinion polls have measurement errors. Another reason is that the theory is limited. It considers only the effect of moral arguments and ignores any effects of singular events, such as statements by the political elite [13] and supreme court decisions [14]. However, whereas generally acceptable moral arguments are assumed to exert a constant force on public opinion, the impact of singular events are limited in time. Given sufficient time to work, the accumulated impact of moral arguments may dominate over the time-limited impact of specific events. It follows that when we use moral arguments to forecast opinion shifts, we should be able to forecast shifts over longer time spans with greater accuracy than shifts over short time spans.

To assess the predictive ability of the moral argument theory, we will compare the accuracy of its predictions with the accuracy of atheoretic predictions based on historical data. Previous research has shown that opinion change in the US often shows long-lasting trends [15,16]. In practice, projection of historical trends into the future could therefore be a feasible approach to forecasting public opinion. From a theoretical point of view, projection of trends makes sense only under the assumption that there is some constant force that determines the speed and direction of opinion change in the long run. The moral argument theory proposes that generally acceptable arguments constitute such a force. Thus, a side effect of the theory is that it legitimizes forecasting by projection of historical trends. However, if the theory is valid, it should be possible to do even better forecasts. Namely, historical trends will always suffer from noise due to sampling error and due to events that have time-limited impact on public opinion. Instead, we can calculate forecasts directly from available measures of how generally acceptable arguments are used to justify different opinions on specific issues. If these measures are sufficiently precise (and assuming that the theory is valid), these forecasts should improve upon forecasts based on historical trends. An additional advantage of forecasting using argument measures is that it allows forecasts even for moral issues for which no historical data are available.

The rest of this paper is organized as follows. First, we describe how the argument advantage of a moral opinion is measured. Then we describe how these measures can be used to forecast shifts in public opinion. For comparison, we present two atheoretic forecasting methods based on historical data. We then assess these methods by examining how well they would have predicted public opinion in 2018 if the methods had been used in 2010, 2012, 2014 and 2016. We find that forecasts based on argument advantage measures are superior. Finally, we present the predictions for 2020 and beyond generated by this forecasting method, and we describe how these predictions will be assessed.

### 1.1. Measuring the argument advantage of a moral opinion

The method we use for measuring the argument advantage of a moral opinion has been described in detail in prior work [5,11]. It relies on a list of generic moral arguments derived from moral foundations research [9]. The list includes both arguments of generally accepted kinds (e.g. harm, fairness and liberty) and arguments of kinds that are less acceptable (e.g., authority and purity). Each kind is represented by three generic arguments. For example, fairness kind is represented by the generic arguments *someone acts unfairly*, *some people are treated differently from others,* and *someone is denied his or her rights.* The full list of generic arguments is given in electronic supplementary material, table S2. To measure the argument advantage for a specific moral opinion *M* (e.g. that the death penalty should be allowed), participants are asked a moral question (e.g., ‘Do you favour the death penalty for persons convicted of murder?’) with a dichotomous response scale (yes/no). They are then presented with the list of generic arguments. From this list, participants tick those arguments they think apply to justify their opinion and, separately, they also tick those arguments they expect are used to justify the opposite opinion by those who hold it. See electronic supplementary material, figure S1, for an example of what the question looks like to respondents. The argument advantage of moral opinion *M* is calculated with respect to generally accepted kinds of arguments. For each of those arguments, we calculate the difference between the proportion of participants who chose the argument to justify *M* and the proportion who chose it to justify *Not M.* The average difference across all arguments of generally accepted kinds is our measure of *M*'s argument advantage. The resulting scores lie between −1 and 1, where a negative value signifies that the opposite opinion, *Not M*, holds the advantage.

Using this procedure, the argument advantage of 98 moral opinions covered by the General Social Survey (GSS) [17] was estimated by Vartanova and colleagues [11]. Importantly, even though the argument advantage measure of an opinion is a subjective measure aggregated across many respondents, it was found to be robust across demographic groups. Thus, we obtain essentially the same argument advantage measure for a given opinion whether we ask women or men, young or old, educated or uneducated, and even whether we ask those who agree with the opinion or those who disagree with it.

Below we make predictions for 102 opinions that were asked in the GSS during the last 10 years. For 75 of them, we use existing measures of argument advantage obtained by the method we have described [11]. The argument advantages of the remaining 27 opinions had not been measured before. To obtain measures we used the same method: each of the 27 items were judged by between 98 and 110 participants from the United States, recruited through Prolific. The mean age of participants was 36.0 years (*SD* = 12.5), 59% of responses were from women. The raw data are accessible at https://github.com/irinavrt/predict-gss-2020.

### 1.2. Forecasting shifts of public opinion on moral issues

In this section, we will develop three models for forecasting moral opinion. The first one is based on the assumption that there is no predictable direction in opinion change and should be considered the null model. The second one assumes there is an underlying force that determines how opinions change. That is, we should assume that public opinions will keep moving in the same direction and speed as in the past. Finally, the third model is based on the argument theory for moral opinions and therefore assumes that argument advantage determines the direction and speed of opinion change. These models will be tested against historical data in the next section. In the final section they will be used to derive actual predictions about future opinions.

As the popularity of an opinion can never decrease below zero and never increase above 100%, we use logit models. For a popularity score *y* between 0 and 1, its logit score is given by logit(*y*) = log(y/(1 − *y*)). Absent a theory for directed opinion change, all opinion changes will be regarded as random events that may go in either direction. This yields the benchmark model

1.1 $$\mathrm{logit}(y_{i,t})=\mathrm{logit}(y_{i,t-1})+\varepsilon_{i,t},$$

where *y_i_*_,*t*_ is the popularity of opinion *i* at time *t* and *ε_i_*_,*t*_ is the random change at this time step, which is assumed to be drawn from a distribution with a mean of zero.

**Figure 1.**
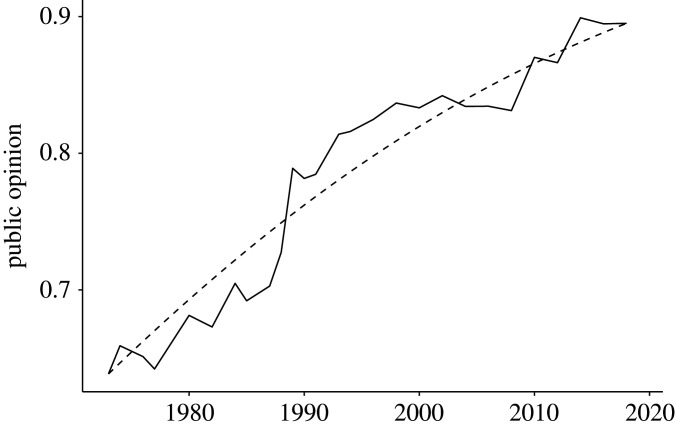
Public opinion change generated by equation (1.2) with a constant positive drift (*c_i_* = 0.03). The dashed line is generated by a model with zero error, the solid line is generated by a model with errors drawn independently from a normal distribution with mean of 0 and variance of 0.019 (*M* = 0 and *s*^2^ = 0.019).

If there is some constant underlying process driving public opinion change in a certain direction, we would instead expect a consistent drift in opinion popularity:

1.2 $$\mathrm{logit}(y_{t})=\mathrm{logit}(y_{t-1})+c_{i}+\varepsilon_{i,t},$$

where *c_i_* is the constant change (in logit units) per time unit caused by the underlying process. The dashed line in figure 1 shows the resulting smoothly increasing opinion change that equation (1.2) results in if there are zero errors and *c_i_* > 0. In the presence of errors, the outcome will be a jagged version of the same graph, which may look like the solid line in figure 1. Note that short-term change is sometimes negative, but the increasing trend is still evident over longer time spans.

We will assume that there is some underlying process driving public opinion change in a certain direction, so that equation (1.2) is a correct model. To make forecasts we need to estimate the drift parameter *c_i_* for a given moral opinion *i.* One approach to estimating the drift parameter is to fit the model in equation (1.2) to the historical opinion data for each opinion *i* up to a time point *T*. We denote the parameter estimates obtained in this way by $c_{i,T}^{\mathrm{trend}}$. Importantly, due to the random noise in historical opinion data (captured by the error term in equation (1.2)), we cannot expect $c_{i,T}^{\mathrm{trend}}$ to be a perfect estimate of the true value of *c_i_*.

We will now assume the underlying process that drives public opinion change in equation (1.2) is in fact the opinion's argument advantage as outlined in our theory. To estimate the drift parameter, we can then take advantage of the existence of survey measures of the argument advantage *AA_i_* of opinion *i*, which are obtainable from surveys [11]. To convert argument advantage scores into values of the drift parameter, we assume a linear relationship:

1.3 $$c_{i}^{}\;=\;\alpha \;+\;\beta AA_{i},$$

Let *α_T_* and *β_T_* be the estimated values for the parameters *α* and *β* that we obtain by fitting a linear relationship between $c_{i,T}^{\mathrm{trend}}$ (as an estimate of the unobservable *c_i_*) and the argument advantage *AA_i_* across many moral opinions *i*. While the trend estimate for each specific issue is error-prone, this approach capitalizes on the availability of such estimates for multiple issues to obtain more reliable estimates of the parameters *α* and *β*. For any specific opinion *i*, we can then obtain a new estimate for its drift parameter, $c_{i,T}^{AA}\;$, by plugging these estimated parameter values together with the argument advantage *AA_i_* of opinion *i* into equation (1.3) as follows:

1.4 $$c_{i,T}^{AA}\;=\alpha_{T}\;+\beta_{T}\;AA_{i}.$$

If the theory that opinion trends are driven by argument advantage is correct, $c_{i,T}^{AA}\;$ may be a better estimator of the drift parameter than $c_{i,T}^{\mathrm{trend}}\;$ is, as the latter uses only data on historical trends up to time point *T*, while the former additionally uses direct measures of argument advantage.

Using the approaches outlined in this section, we obtain three different predictions for public opinion at target time *T* + *τ* given opinion data up to time *T,* the starting point of the forecast. The benchmark prediction is obtained by

1.5 $$y_{i,T+\tau }^{\mathrm{benchmark}}=y_{i,T}.$$

The trend-based prediction is

1.6 $$y_{i,T+\tau }^{\mathrm{trend}}={\mathrm{logit}}_{}^{-1}(\mathrm{logit}(y_{i,T})+c_{i,T}^{\mathrm{trend}}\;\tau ).$$

The argument advantage-based prediction is

1.7 $$y_{i,T+\tau }^{AA}={\mathrm{logit}}_{}^{-1}(\mathrm{logit}(y_{i,T})+c_{i,T}^{AA}\;\tau ).$$

Our hypotheses are that trend-based predictions are overall more accurate than benchmark predictions and that argument advantage-based predictions are even more accurate overall.

### 1.3. Assessment of the forecasting models using existing data

Following previous work on the moral argument theory [5], we focus on moral opinions that are included in the GSS. This survey measures public opinion on a wide range of moral issues every 2 years. At the time of this registered report, the most recent available data are from the 2018 wave. We will here assess how well our forecasting models are able to predict public opinion in 2018 using opinion data from previous years as well as data on argument advantage.

#### 1.3.1. Selection of moral issues

Prior work has measured the argument advantage for 98 moral opinions covered by the GSS [9,12]. The 2018 wave of the GSS included 63 of these items. We focus on these 63 items.

#### 1.3.2. Calculation of the popularity of each opinion in each wave

To quantify opinion popularity as a percentage of the population, we dichotomized all items by omitting neutral responses and by combining graded responses (e.g. all nuances of ‘agree’ versus all nuances of ‘disagree’). On these dichotomous data, we use the sampling weights provided by the GSS to estimate the percentage of the population who agreed with an opinion in each wave.

#### 1.3.3. Starting points of forecasts

To assess the accuracy of forecasts made at different points in time, we predict public opinion at target time 2018 using data from either 8, 6, 4 or 2 years in advance. This means that, to make the predictions, we use all GSS data up to the starting points 2010, 2012, 2014 and 2016, respectively.

#### 1.3.4. Calculation of forecasts

Forecasts are calculated using the methods described in §1.2. Thus, for each starting point *T*, and each opinion *i*, we first obtain a value for $c_{i,T}^{\mathrm{trend}}\;$ by ordinary least squares (OLS) fitting the model in equation (1.2) to the historical opinion data up to, and including, the starting point. We then obtain values *α_T_* and *β_T_* by fitting a linear relationship between $c_{i,T}^{\mathrm{trend}}$ and the argument advantage *AA_i_* across all 63 moral opinions *i*. These parameter values are reported in table 1.

**Table 1.** 2018 point forecast accuracy assessed by the mean squared deviation (MSD) between predicted and observed public opinion in 2018. Note: the last two columns report differences with bootstrapped 95% confidence intervals based on 500 draws capturing the uncertainty caused by sampling error in observed public opinion.

starting point (*T*)	parameter values *α_T_*, *β_T_* used for predictions from AA	MSD_AA_	MSD_Benchmark_	MSD_Trends_	MSD_Benchmark_ − MSD_AA_	MSD_Trends_ − MSD_AA_
2010	0.01, 0.23	19.9	44.9	27.8	25.0 [19.4, 30.3]	7.9 [4.3, 11.1]
2012	0.01, 0.22	25.1	44.7	27.8	19.6 [15.5, 23.5]	2.7 [0.1, 5.2]
2014	0.01, 0.23	15.5	22.9	18.3	7.4 [4.7, 10.0]	2.8 [1.1, 4.3]
2016	0.01, 0.23	6.4	8.6	6.5	2.2 [0.8, 3.5]	0.0 [-0.8, 0.7]

Using equation (1.4) we then obtain values $c_{i,T}^{AA}$. For each starting point *T* we use equations (1.5), (1.6) and (1.7) to make three sets of predictions (benchmark, trends and AA) for the popularity of the 63 moral opinions at the target time *T* + *τ* = 2018.

#### 1.3.5. Assessing the accuracy of forecasts

To assess the accuracy of a forecast we compare the predicted popularity in 2018 with the observed popularity in 2018 for all 63 moral opinions. The metric we use for comparison is the mean squared deviation (MSD). This means that, for each opinion, we first calculate the difference (in percentage points) between the predicted value for 2018 and the observed value for 2018; we then square these numbers and calculate their mean across all 63 opinions. Table 1 presents the MSD for each prediction method (AA-based, Benchmark and Trends-based) and each starting point (2010, 2012, 2014 and 2016).

Note that forecasts based on projection of historical trends were consistently more accurate than benchmark forecasts. This means that the drift in public opinion tends to have a consistent direction. Historical opinion trends, therefore, contain information about how public opinion will develop in the future. According to the moral argument theory, this is because opinion trends are caused by some opinions holding an argument advantage. Consistent with this theory, AA-based forecasts were even more accurate than trends-based forecasts. The last two columns of table 1 say how much better (in the MSD metric) AA-based forecasts were than the alternative forecasts, with 95% confidence intervals capturing the uncertainty caused by sampling errors in the observations of public opinion in 2018.^1^ Note that AA-based forecasts were clearly superior to other forecasts when predictions were made at least 4 years in advance. When forecasting across shorter time spans, opinion changes are smaller and measurement errors, therefore, play a larger role, increasing the relative uncertainty.

**Figure 2.**
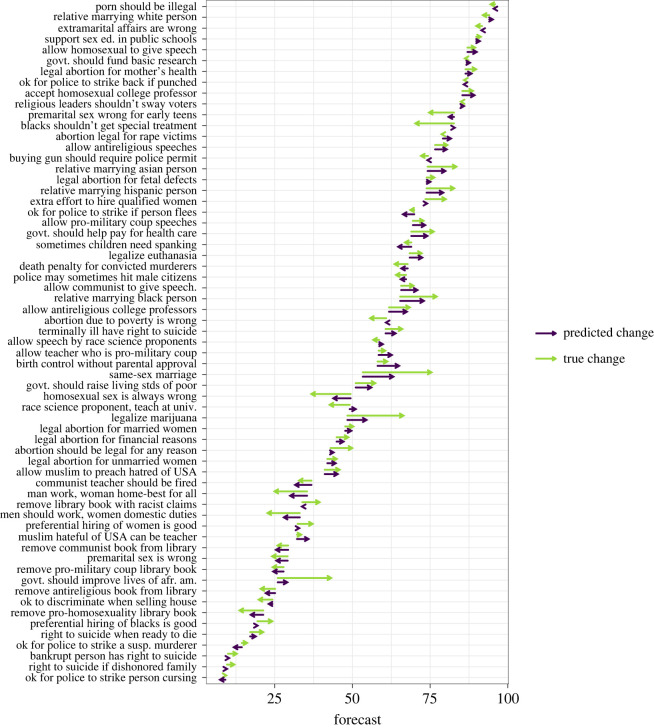
The change in the popularity of 63 moral opinions in the United States from 2010 to 2018 as observed in GSS polls (green arrows) and as predicted from argument advantage measures (purple arrows). The items are abbreviated in the figure. For the full text of items, see electronic supplementary material, table S1.

Figure 2 gives an illustration of how the AA-based forecast of opinion changes from 2010 to 2018 compared with the observed opinion change for each of the moral opinions we study.

## 2. Predictions

The data from the 2020 wave of the GSS are expected to be released in October 2021. In advance of this data release, we here make predictions on how public opinion will have shifted since the latest available wave for that issue. We make predictions for 102 issues that have been asked at least once since 2010. These include the 63 items that we used in the analysis of forecasting methods in §1. The remaining 39 items were not included in that analysis either because they were not asked in 2018 or because they have not been included sufficiently many times for trends to be estimated.

**Figure 3.**
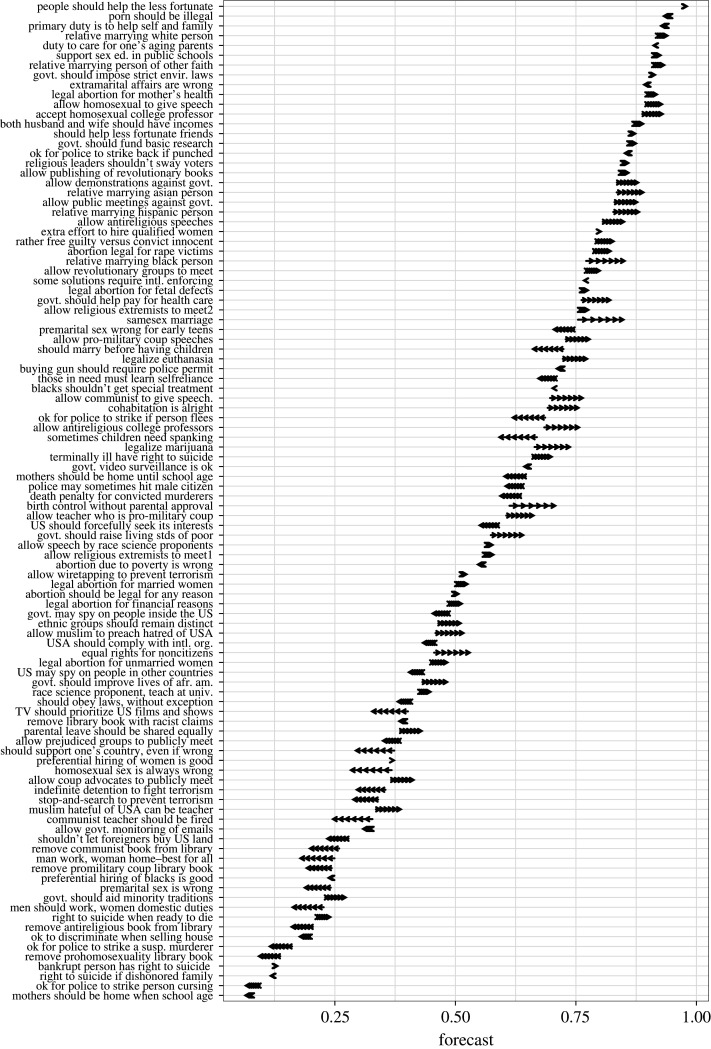
The predicted change in the popularity of 102 moral opinions in the United States from 2018 to 2020, 2022, 2024, 2026, 2028 and 2030. More details are found in electronic supplementary material, table S1.

As the starting point *T* of the forecast for a given issue, we use the latest available year for the issue (i.e. *T* = 2018 for most issues but not all). For each of the 97 opinions *i* that were measured at least twice so that a trend can be estimated, we first obtain a value for $c_{i,T}^{\mathrm{trend}}\;$ by fitting the model in equation (1.2) to the historical opinion data up to, and including, *T*. We then obtain parameter values *α*_2018_ and *β*_2018_ by fitting a linear relationship between $c_{i,T}^{\mathrm{trend}}$ and the argument advantage measures *AA_i_* across the 97 opinions in the subset (*α*_2018_ = 0.00 and *β*_2018_ = 0.24).^2^ Using equation (1.4), we then obtain values $c_{i,2018}^{AA}$ for all 102 opinions for which we have argument advantage measures. For each target point *T* + *τ* = 2020, 2022, 2024, 2026, 2028 and 2030, we use equation (1.7) to make AA-based predictions for the popularity of the 102 moral opinions. These predictions are illustrated in figure 3. They are given in numerical format in electronic supplementary material, table S1 and figure S2 illustrates the predictions in another way, with prediction intervals.

## 3. Assessing the accuracy of the forecast for 2021

### 3.1. Methods

The assessment was carried out as described in the preregistered report (https://osf.io/ax8wt). Following the preregistered report, we were to assess, upon the release of the 2020 GSS data, the accuracy of our argument advantage (AA)-based forecast for 2020 exactly as we did above for 2018, and as explained in the preregistration:

(1) The benchmark prediction for all 102 issues was to be calculated as in §1.3.4. For the 97 issues for which good historical trends could be calculated, we were to additionally calculate the prediction from projecting the historical trend as in §1.3.4.
(2) For all issues that are measured in GSS 2020, out of the 102 included in our forecast, the observed 2020 popularity of the corresponding opinion was to be estimated as described in §1.3.2.
(3) The accuracy of our forecast was to be assessed as in §1.3.5, that is, by calculating how the mean squared deviation (MSD) to observed public opinion in 2020 differs between the forecasts, taking the uncertainty due to sampling error into account. When comparing the MSD between AA-based and trends-based forecasts, only the 97 issues for which the latter is defined were to be used.

We defined our main success criterion as the comparison with the benchmark forecast. Our theory will have succeeded at predicting future opinion change if observed public opinion in 2020 is closer (as measured by MSD) to the AA-based forecast than to the benchmark forecast.

If AA is the driver behind trends, the AA-based forecast should also outperform the trends-based forecast. However, due to sampling error in observed public opinion, this difference is unlikely to be detectable when forecasting over such a short time span as 2 years (table 1). We expected the difference in accuracy between AA-based and trends-based forecasts to be detectable in 2022 and onwards.

#### 3.1.1. Change of prediction target year from 2020 to 2021

Due to the COVID-19 pandemic, the data collection for the 2020 wave of the GSS could not be conducted as originally planned. The collection was delayed by a year. Instead of being gathered during the spring of 2020, the data was collected between December 2020 and May 2021. The released data are therefore referred to as the 2021 GSS by the GSS organization. To assess the accuracy of the prediction methods, we will therefore compare public opinion as observed in the 2021 GSS data with what each method would have predicted for 2021 rather than 2020. Specifically, this means that trends-based and AA-based predictions of change are revised to be 50% larger in logit points. Benchmark predictions are not affected at all.

#### 3.1.2. Lower quality data

In addition to the delay, COVID-19 also caused a major change in the data collection method. For detailed information on the 2021 GSS methodology and its implications, we refer to https://gss.norc.org/Get-The-Data. In brief, the data collection method changed from the traditional in-person interviews to a mail-to-web method. As a result, there was a huge drop in the response rate, from 60% for the 2018 GSS to just 17% in the 2021 GSS. Researchers must, therefore, be aware that observed changes in public opinion may be influenced by the stronger selectivity of the sample.

#### 3.1.3. Special political events

The last 2 years have been unusually volatile in US politics, with two events standing out as especially likely to cause shifts in public opinion. First, there were nationwide protests following the murder of George Floyd. It has already been documented, in a dataset collected weekly, that this led to a shift in opinion about the police and about the discrimination of African Americans [18]. Second, the US went through a contested election that ended with a mob of supporters of President Donald Trump attacking the United States Capitol. This momentous political event is likely to have made people reassess the risks involved in allowing people to advocate for coups. Note that such singular events are likely to cause an immediate shift in certain moral opinions but cannot explain long-term trends. When assessing our predictions of opinion change below, we will complement the preregistered method of assessment by an analysis where we exclude items that connect with the above-mentioned singular events.

### 3.2. Results

Calculation of the predictions for 2021 and the assessment of the forecast accuracy followed the preregistered protocol. Out of the 102 items for which we provided predictions, 60 items were included in the 2021 GSS study.^3^ For these 60 items, we calculate AA-based, Trends-based, and Benchmark predictions forecasts for 2021, as described in §1.3.4. Electronic supplementary material, table S3 reports the forecasts and the public opinion observed in the 2021 GSS data. The mean squared deviation between the forecasted and observed public opinion is reported in table 2. The AA-based predictions significantly outperformed the benchmark. Thus, our prediction method is deemed a success according to our assessment criterion. AA-based predictions performed similarly to the Trends-based predictions.

**Table 2.** 2021 point forecast accuracy, assessed by the mean squared deviation (MSD) between predicted and observed public opinion. Note: the last two columns report differences with bootstrapped 95% confidence intervals based on 500 draws, capturing the uncertainty caused by sampling error in observed public opinion.

MSD_AA_	MSD_Benchmark_	MSD_Trends_	MSD_Benchmark_ − MSD_AA_	MSD_Trends_ − MSD_AA_
29.7	32.7	29.7	2.9 [1.2, 4.7]	0.0 [−0.8, 0.9]

#### 3.2.1. Additional results (not preregistered)

Comparing table 2 with table 1, it is striking that all prediction methods performed considerably worse for the 3-year span from 2018 to 2021 than for the 4-year span from 2014 to 2018. In particular, the larger MSD for the benchmark method shows that observed opinion changes were overall larger from 2018 to 2021. To some extent, this will be an artefact of the change in data collection method, which we cannot control for. However, to some extent the large shifts in observed public opinion may be caused by the singular political events discussed above. If so, predictions should be especially off for items that connect with these events. We examined this in an additional, not preregistered, analysis in which we group the 60 items into 21 themes (see the ‘group’ column in electronic supplementary material, table S3). Themes were chosen narrowly based on the item's subject matter. For instance, questions about free speech were separated into categories depending on the target of the speech, so that we can look specifically at shifts in opinions about free speech for people arguing for a military overthrow of the government. Table 3 shows that the accuracy of the forecasts varied substantially across themes. In line with the hypothesis that singular events caused opinion shifts, the MSD was especially high for freedom of speech for militarists and racists as well as for societal treatment of African Americans and police violence. These four themes comprise 14 items. Exclusion of these 14 items reduces the MSD considerably for all three prediction methods, but AA-based predictions are now significantly better than Trends-based predictions and remain better than Benchmark (table 4).

**Table 3.** 2021 point forecast accuracy for different issue groups sorted by the MSD for the Benchmark forecast.

groups	MSD_AA_	MSD_Benchmark_	MSD_Trends_
free speech militarist	160.8	128.2	157.0
societal treatment of African Americans	60.2	65.6	48.3
free speech racist	66.0	60.4	57.9
police violence	39.8	43.2	31.0
welfare	25.3	40.4	43.8
prosexuality	26.2	36.6	30.6
abortion	29.3	35.4	33.1
homosexuality	3.7	14.9	5.5
societal treatment of women	8.2	10.2	15.4
racial marriage	1.9	5.5	3.3
free speech homosex	4.3	5.1	4.2
free speech atheist	5.8	4.8	4.9
suicide	5.1	4.6	3.7
free speech communist	0.5	4.4	1.0
free speech muslim	7.9	2.2	7.8
fund science	0.1	0.0	1.5
single items			
spanking	93.7	133.6	92.7
grass	30.4	55.5	19.4
capital punishment	33.8	47.1	44.4
gun law	40.1	43.5	43.1
racial discrimination	0.1	0.9	1.1

**Table 4.** 2021 point forecast accuracy after exclusion of 14 items connected to singular events. Note: the last two columns report differences with bootstrapped 95% confidence intervals based on 500 draws, capturing the uncertainty caused by sampling error in observed public opinion.

MSD_AA_	MSD_Benchmark_	MSD_Trends_	MSD_Benchmark_ − MSD_AA_	MSD_Trends_ − MSD_AA_
15.7	21.3	18.3	5.6 [3.5, 7.7]	2.5 [1.6, 3.5]

Finally, we provide some other metrics to give a better understanding of how much of the opinion change we succeeded in predicting. One such metric is how often the direction of change was correctly predicted. AA-based predictions were correct about the direction of change for 68% of items, or 76% after the exclusion of 14 items connected to singular events. The corresponding success rates for Trends-based predictions were 60% and 61%, respectively. These numbers should be compared with the 50% success rate expected from random guessing. (Recall that Benchmark predictions are that there will be no change in any direction.)

In addition to getting the direction of change correct, we would like to get the size of the change right. We can then look at the proportion of variance (R2) in the estimated changes that is explained by our predictions. For AA-based predictions, the proportion of variance explained is 13 or 39% after exclusion of the 14 items. The corresponding proportions for Trends-based predictions were 11 and 15%, respectively. Note that the estimated change has considerable measurement errors that will bias these proportions downwards.

## 4. Discussion

Based on the moral argument theory of Eriksson & Strimling [4] we developed pinpoint predictions for how moral opinion will change on 102 issues. These predictions are based on measures of the kinds of arguments that justify each opinion (together with parameters capturing the general speed of change, estimated from historical data). As our main success criterion, we compared the accuracy of our prediction with a benchmark prediction of no change. Our prediction was significantly better. Thus, this study supports the general notion that opinion change is to some extent predictable as well as the specific theory that such predictions can be based on measures of the kinds of moral arguments that justify specific opinions. A consequence of the moral argument theory is that public opinion on moral issues will exhibit long-term directed trends. In line with this, we found that projection of historical trends is also superior to benchmark predictions. However, our analyses indicate that projection of trends is less accurate than using argument advantage measures to predict opinion change over longer time spans.

Several features made 2020–2021 an extraordinary year for any attempt to predict public opinion. For one thing, the COVID-19 pandemic made the GSS change the data collection method from face-to-face interviews to an online survey, which resulted in a very large drop in the response rate and, consequently, a less representative sample of respondents. For another, the occurrence of two singular political events, the George Floyd riots and the January attack on the Capitol, may have caused immediate opinion shifts. In support of the latter notion, we found that opinion shifts tended to be especially large for items that connect to these singular events, that is, items about how society treats African Americans, police violence and free speech surrounding racism and coups. Exclusion of this subset of items led to a substantial improvement of the accuracy of our predictions.

The finding that projection of historical trends yields better predictions than benchmark is a clear indication that there must exist *some* mechanism driving opinions in a certain direction, as this is the only way previous trends can inform us about the future. The success of the predictions based on the moral argument theory indicates *what* mechanism this is: an opinion will tend to spread if it has an advantage over the opposite opinion with respect to being justifiable by generally acceptable kinds of arguments. The larger the argument advantage is, the faster the opinion tends to spread. Note that our theory allows us to predict future opinion change even on issues for which there is no historical opinion data. All we need is to measure which opinion on the issue has the argument advantage and how big the advantage is. The method is also not specific for the United States. In any country where data on opinion change over time are available, the general speed of change (*β*) could be estimated and the same prediction method could then be employed. Thus, our theory provides a method for prediction opinion change that is more generally applicable than the trends-based method.

While our theory was overall quite successful at predicting opinion change, it performed poorly at predicting opinion shifts on certain issues that were connected to a couple of singular political events. Ours is a theory about a mechanism that generates long-term opinion trends. It cannot predict shifts in specific opinions that are caused by events outside the theory. We acknowledge that there are other theories about how underlying conditions in the US right now increase political violence [19], but it seems unlikely that such theories could have predicted the exact timing and type of events that occurred. In the short term, such events may have more impact than the mechanisms for gradual directed opinion change that our theory is based on. Nonetheless, if the argument theory is correct about gradual directed opinion change being driven by the nature of moral arguments, its predictive power will increase over longer time periods in which the effect of impactful events becomes small compared with the accumulated change that is due to the argument mechanism.

Overall, this study shows that moral opinion change can to some extent be predicted, even under unusually volatile circumstances. Note that the prediction method used in this paper is quite rudimentary. Specifically, the method is only based on a very simple survey measure of each opinion's argument advantage and the use of historical opinion data to calibrate a parameter for converting such measures to predicted change rates. Given that the direction is predicted completely based on surveys about argument advantage it is remarkable that the direction was correctly predicted in two-thirds of the cases (three-quarters if the issues related to singular events were excluded). Even so, the method can probably be improved. We challenge the public opinion research community to find ways to further improve how future opinion changes can be predicted and to test such predictions in the United States as well as in other countries with sufficiently rich data on moral opinions. As a new benchmark, we have spelled out the predictions our method produces (based on historical opinion data that was gathered up until 2018) for how US public opinion on moral issues will change until 2028. If anyone can devise a method that makes more accurate predictions, without using more recent data, this will improve our understanding not only of future opinion change but also of its causes.

## Data Availability

Data and relevant code for this research work are stored in GitHub: https://github.com/irinavrt/predict-gss-2020 and have been archived within the Zenodo repository: https://doi.org/10.5281/zenodo.5799479.

## Footnotes

1 For opinion *i*, let Ob*_i_* denote the observed value of public opinion in data for 2018 and let A*_i_* and B*_i_* denote the predicted values from the AA-based and Benchmark forecasts. The observed superiority of A over B is the difference MSD(Ob,B) − MSD(Ob,A). To assess the uncertainty in this quantity, we express Ob*_i_* as Tr*_i_* + e*_i_*, where Tr*_i_* is the true value in the population and e*_i_* is the sampling error. The error in the observed superiority can then be expressed by the formula [MSD(Tr,B) − MSD(Tr,A)] − [MSD(Ob,B) − MSD(Ob,A)] = Mean(2e_i_(B*_i_* − A*_i_*)), where the mean is taken over all opinions *i*. Confidence intervals are obtained using the 2.5 to 97.5 percentile interval for this error in 500 bootstrap samples.

2 In the preregistered version, these parameter values were incorrectly calculated to be 0.01 and 0.22, respectively, due to a coding error: for the GSS items wrkbaby and wrksch, the AA had accidentally been coded as 0.15 instead of −0.15 (see electronic supplementary material, table S1). When this error is corrected, our method produces the corrected parameter values 0.00 and 0.24 instead. Because the role of these parameter values is to determine how to convert AA values to predictions of opinion change, a small correction of the parameter values automatically implies a small but systematic correction of all our predictions. In this paper it is the corrected predictions that we present.

3 For some of the long-running items in the GSS, the 2021 wave included more than one version for various reasons. In these cases we have selected the version that is closest to the original.
